# Prospective Registry Study on Thermal Liver Ablation of Primary and Secondary Liver Tumours Named the A-IMAGIO Study

**DOI:** 10.1007/s00270-025-04093-9

**Published:** 2025-06-23

**Authors:** A. L. van der Velden, C. A. M. Verhagen, F. Gholamiankhah, H. Rahmani, R. M. van Dam, J. J. van Duijn-de Vreugd, S. R. Simon, P. Hendriks, G. C. M. van Erp, R. R. M. M. Knapen, L. Volmer, K. Overduin, J. P. B. M. Braak, R. Bale, G. Laimer, R. Lanocita, M. R. Meijerink, Y. Kampfer, A. Denys, P. Littler, B. Sternberg, H. Kobeiter, M. L. J. Smits, M. J. L. van Strijen, K. J. Pieterman, A. Broersen, J. Dijkstra, R. Brecheisen, M. C. Burgmans, C. van der Leij

**Affiliations:** 1 Department of Radiology and Nuclear Medicine, Maastricht University Medical Center+, Maastricht, The Netherlands; 2 GROW, School for Oncology and Reproduction, Maastricht University, Maastricht, The Netherlands; 3 Department of Radiology, Leiden University Medical Center, Leiden, The Netherlands; 4 Department of Surgery, Maastricht University Medical Center+, Maastricht, Netherlands; 5 CARIM, School for Cardiovascular Diseases, Maastricht University, Maastricht, The Netherlands; 6 Department of Radiology, Radboud University Medical Center, Nijmegen, The Netherlands; 7 Department of Surgery, Leiden University Medical Center, Leiden, The Netherlands; 8 Department of Radiology, Medical University Innsbruck, Innsbruck, Austria; 9 Department of Radiology, Department of Radiology, Fondazione IRCCS Istituto Nazionale dei Tumori di Milano, Milan, Italy; 10 Department of Radiology, Amsterdam University Medical Center, Amsterdam, The Netherlands; 11 Institute for Health Economics and Clinical Epidemiology, Faculty of Medicine and University Hospital Cologne, University of Cologne, Gleueler Straße 176-178, 50935 Cologne, Germany; 12 Department of Radiology and Interventional Radiology, CHUV University of Lausanne, Rue du Bugnon, Lausanne, Switzerland; 13 Departments of Interventional Radiology, Freeman Hospital, Newcastle Upon Tyne, UK; 14 Radiology Department, H. Mondor Hospital, Assistance Publique-Hôpitaux de Paris, University Paris Est Creteil, Creteil, France; 15 Department of Radiology and Nuclear Medicine, University Medical Centre Utrecht, Utrecht, The Netherlands; 16 Department of Radiology, St. Antonius Hospital, Nieuwegein, The Netherlands; 17 Department of Radiology, Erasmus Medical Center, Rotterdam, The Netherlands; 18 Division of Image Processing, Department of Radiology, Leiden University Medical Center, Leiden, The Netherlands

**Keywords:** Hepatocellular carcinoma, Colorectal liver metastases, Thermal ablation, Microwave ablation, Radiofrequency ablation, Artificial intelligence, Deep learning

## Abstract

**Purpose:**

The long-term objective of the Ablation-IMaging and Advanced Guidance for workflow optimization in Interventional Oncology (A-IMAGIO) project is to develop a standardized, accessible, low-complex, high-precision, end-to-end solution for treatment planning, needle guidance, and treatment evaluation for thermal liver ablation.

**Materials and Methods:**

This is a prospective, international, multicentre, observational registry study. Patients will be included with age ≥ 18 years, diagnosed with primary or secondary liver tumours, and undergoing thermal liver ablation. A detailed dataset of medical history, baseline clinical and imaging parameters, tumour characteristics, ablation technique/parameters, treatment outcomes, periprocedural images, and adverse events, will be collected for all participants. This data will be used to develop AI algorithms for prognostic modelling and quantitative imaging analysis. Additionally, costs associated with thermal liver ablation clinical pathway will be evaluated.

**Expected Gain of Knowledge:**

The results of this registry study are expected to provide profound insight in current variability among centres in performing thermal liver ablation, and identify best practices in order to eventually facilitate standardization and universally excellent clinical outcomes.

**Trial Registration:**

National Institute of Health Clinical trial database (NCT06179602) https://clinicaltrials.gov/study/NCT06179602.

**Graphical Abstract:**

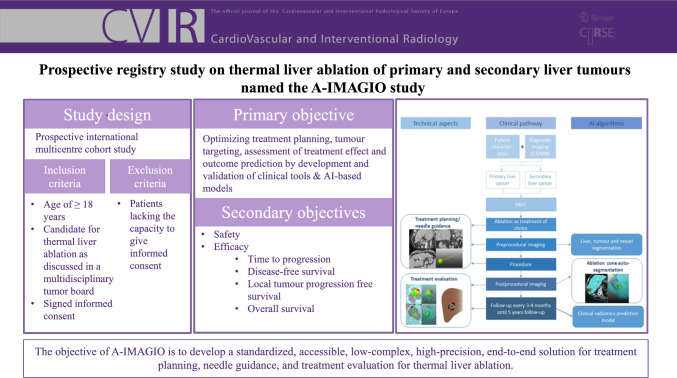

**Supplementary Information:**

The online version contains supplementary material available at 10.1007/s00270-025-04093-9.

## Introduction

For both primary and secondary liver tumours, thermal liver ablation (TLA) (i.e. RFA and MWA) has proved to be an effective minimally invasive treatment, associated with a low complication rate [1–4]. According to the Barcelona Clinic Liver Cancer (BCLC) staging system, percutaneous TLA is recommended as first-line therapy in patients with very early-stage (0) and early-stage (A) hepatocellular carcinoma (HCC) by the European Association for the Study of the Liver (EASL) and European Society of Medical Oncology (ESMO) guidelines [5, 6]. In oligometastatic colorectal liver metastases (CRLM) up to 3 cm, overall survival (OS) after TLA is non-inferior to surgical resection while offering the advantage of lower complication rates and shorter hospital admissions [7]. Despite the growing evidence demonstrating the efficacy and safety of TLA, there is still a certain resistance to incorporate TLA into clinical guidelines as the treatment of first choice. This is primarily due to scarcity of large-scale high-quality clinical trials comparing TLA to surgical resection. For secondary liver tumours other than CRLM, outcomes of TLA have only been documented in case reports, case–control studies, and retrospective studies, with an absence of large-scale prospective data [8–11].

Alongside the more prominent application of TLA, a wide variety of advanced software tools has become available to improve treatment planning, tumour targeting, and assessment of immediate and long-term treatment response. In recent years, artificial intelligence (AI) models for planning and evaluation have gained interest, as they can aid in improving workflows in different clinical settings. Although these technical advancements were expected to deliver consistent outcomes with minimal operator dependency, they are still not widely used and have paradoxically led to greater diversity in workflow practices [12, 13]. The consequence of this treatment and workflow variability imposes the risk of being followed by inconsistency in clinical outcomes among treating physicians and centres. This variability hampers implementation of TLA as standard of care. For this reason, the European Union funded, A-IMAGIO (Ablation-IMaging and Advanced Guidance for workflow optimization in Interventional Oncology) project was initiated. One of the main goals of the A-IMAGIO project is to expedite the adoption and acceptance of multimodality ablation therapy as treatment of first choice for primary and secondary liver tumours by optimizing treatment workflow and reducing treatment variability.

Within the A-IMAGIO study, data are collected from various centres to gain insight into inter-centre treatment variability and outcomes. This knowledge will be used to identify best practices and techniques and eventually develop a standardized and optimal workflow for TLA.

To support and improve this, clinical and imaging data will be used to develop and validate machine learning and deep learning (DL) based models that allow software-based treatment planning, needle guidance, and assessment of treatment effect.

## Materials and Methods

### Trial Design and Study Setting

This prospective, international, multicentre registry study is coordinated by Leiden University Medical Centre (The Netherlands) and Maastricht University Medical Centre + (The Netherlands). Participating centres include Radboud University Medical Center (The Netherlands), Amsterdam University Medical Centre (The Netherlands), Istituto Nazionale dei Tumori di Milano (Italy), University Hospital of Innsbruck (Austria), Lausanne University Hospital (Switzerland), University Medical Centre Utrecht (The Netherlands), Henri Mondor University Hospital (France), and Newcastle Freeman Hospital (UK). Other centres will be welcome and/or invited to participate.

A detailed overview of the collected data concerning patient and tumour characteristics, ablation technique, and treatment outcomes is described in supplementary 1. Additionally, imaging data will be collected on treatment planning, assessment, and follow-up. To expand the database, retrospective data and data obtained from previously conducted or ongoing prospective studies on TLA of primary and secondary liver tumours will be used [14–16].

### Participants

Table 1 shows the in- and exclusion criteria for participation. For study participation, informed consent will be obtained.

**Table 1:** In- and exclusion criteria

Inclusion criteria	Exclusion criteria
Age of 18 years or above Candidate for percutaneous thermal liver ablation, as discussed in a multidisciplinary tumour board Signed informed consent	Patients lacking the capacity to give informed consent

### Intervention

To provide the opportunity to assess current practice, and techniques, identify areas of heterogeneity and identify best practices, patients will undergo percutaneous TLA as standard of care of the participating centre. The procedure is allowed to be performed under general anaesthesia or deep sedation, and the choice of ablation method (MWA or RFA), apparatus, ablation settings, and image guidance techniques for needle placement is also left to the discretion of the treating physician. All patients will undergo a dual-phase contrast-enhanced (cone-beam) CT scan with at least an arterial and portovenous phase immediately before and after the procedure. Contrast can be administered either intravenously, or through a catheter in the hepatic artery, also known as CT hepatic arteriography. Assessment of technical success (i.e. complete eradication of the tumour with sufficient ablation margin) can be evaluated by visual assessment, image co-registration software, and/or confirmation software. As standard of care, a circumferential minimal ablation margin (MAM) of at least 5 mm is generally aimed for.

### Follow-Up

Follow-up will be conducted according to the standard of care of the participating centre. In general, the follow-up of TLA encompasses physical blood tests and imaging such as (CE)CT, MRI, and/or PET-CT, scheduled at three to 6 months intervals. Patients will be monitored for 5 years following inclusion in the study. Follow-up scans will be reviewed by an experienced radiologist, as per standard of care.

### AI Models Development

#### Ablation Margin Assessment

Accurate segmentation of tumour and ablation zones, along with co-registration of pre- and post-ablation CT scans, is essential for ablation margin assessment, such as calculating the MAM. To automate this process, DL-based segmentation models will be developed using multicentre data, with expert segmentation of the tumours and ablation zones serving as ground truth for model training and validation. Two experienced interventional radiologists will independently segment the tumours and ablation zones on the pre- and post-ablation CT scans, respectively. Subsequently, pre- and post-ablation CT scans will be automatically co-registered by aligning liver contour and/or vessel segmentation masks, with manual adjustments made if needed.

#### Prognostic Model for Early Recurrence

Prognostic models will be developed for prediction of early recurrence following TLA for both primary and secondary liver tumours. Potential predictive imaging features will be identified through radiomics-based and DL-based extraction of contrast-enhanced CT scans, with liver, tumour, and ablation zone segmentation. Clinical factors will be identified by uni- and multivariate cox regression analysis. The potential factors will be evaluated for their predictive value, and the selected prognostic biomarkers will be combined as inputs for the prognostic model.

#### Treatment Planning Model

To determine the optimal trajectory and number of needles required for effective TLA, a planning model will be developed incorporating key tumour and liver tissue characteristics, such as tumour type, size, shape, location, and liver tissue composition. A segmentation model will be used to accurately delineate liver, tumour, and blood vessels, to identify safe needle trajectories. This model will be trained and validated using preprocedural CT scans containing ground-truth annotations for liver, tumour, and blood vessels, as provided by the experienced interventional radiologists mentioned above. Machine learning models will be developed to recommend the most effective ablation settings. These models, using clinical and imaging data from past treatments, will dynamically adjust parameters to achieve the desired ablation zone, enhancing its precision while minimizing risks of damaging healthy liver tissue.

#### Data Handling

Data from the participating centres will be split into training, validation, and test sets to evaluate model performance. The training and validation sets will serve model development, including hyperparameter optimization and iterative refinements, while the test set will be held out to provide an unbiased estimate of the final model accuracy. In addition, an external validation cohort from a separate clinical centre will further assess the generalizability of the models.

### Outcomes

#### Primary Outcomes

The primary outcomes of this registry study are to investigate practice variability and identify best practices, and to develop and validate clinical tools and AI models to optimize treatment planning, tumour targeting, assessment of treatment effect, and outcome prediction in patients undergoing TLA for primary or secondary liver tumours.

#### Secondary Outcomes

Secondary outcomes include safety and efficacy of TLA. Safety will be assessed by monitoring complications, up to 3 months following TLA, according to the CIRSE classification system for complications [17]. Perioperative complications are defined as those occurring up to 1 month following TLA, while delayed complications are those occurring after 1 month. Efficacy will be assessed as disease status at different time points, time-to-progression, disease-free survival (DFS), local tumour progression free survival, and OS according to standard terminology [19, 20]. Other outcomes that will be assessed are resource consumption and total costs associated with the clinical pathway of TLA will be assessed from the hospital's perspective. A micro-costing approach utilizing the time-driven activity-based costing will be conducted [18].

### Sample Size

The sample size was not predetermined, as this is a registry study. However, approximately 1200 patients are expected to be required for development of aforementioned AI algorithms. Prospective inclusions began on January 17th 2024. This project is designed to continuously include new patients providing an ongoing source for studies aimed at optimizing clinical outcomes of TLA.

### Statistical Analysis

Descriptive findings will be summarized with means and standard deviations (SD). Continuous variables will be presented with means and SD or ranges for nonparametric distributions. Categorical variables will be shown as numbers and percentages. Kaplan–Meier estimates will be used to assess local and overall recurrence, DFS, and OS. Logistic and Cox regression analyses will be performed to determine predictors for local and overall recurrence, DFS, and OS. The methods used for feature selection and model generation will be based on the extent of data availability. Data will be analysed using R (version 4.2.1; R Core Team, 2023, Austria). Cost of clinical pathway of TLA will be analysed using IBM SPSS Statistics (version 29, IBM, Armonk, USA).

## Expected Gain of Knowledge

The role of TLA in the management of malignant liver tumours is currently at a pivotal point, supported by recent pioneering studies that demonstrated its non-inferior efficacy compared to surgical resection, along with low complication rates and short hospital stays [7, 21]. Yet, the implementation of TLA as the treatment of choice remains hindered due to discrepancies in the reported oncological outcomes.

Over the past decade, percutaneous TLA techniques have undergone significant advancements leading to improved oncological outcomes [5, 7, 9, 15]. For each distinct phase of the treatment, supportive tools and software have been developed [22–28]. The opposing facet of these technical developments, however, is increased variability in workflows across centres and countries [12, 13]. Considering recent guideline updates, endorsing the application of TLA for primary and secondary liver tumours, an increase in its application and performing centres is anticipated [5, 6]. Therefore, minimizing inter-operator variability by standardization is crucial to certify global reproducibility of excellent clinical outcomes.

The aim of the A-IMAGIO study is to assess which techniques and tools are currently being used and identify areas of heterogeneity and excellence on a multicentre international scale. This collected clinical and imaging data will also be utilized to develop and validate an AI-based prognostic tools on early recurrence. The proposed AI models are anticipated to advance our management of TLA by the integration of automated image analysis. Specifically, the development of deep learning-based segmentation for ablation margin assessment will enhance the precision of tumour and ablation zone delineation, leading to accurate automated margin measurements an objective endpoint to determine technical success. The prognostic models, which will integrate relevant radiomic features and clinical parameters, are expected to yield robust predictors for early recurrence, facilitating a personalized treatment approach.

Collectively, these innovations promise to refine current ablation strategies, and to contribute to the technical standardization of TLA, ultimately designing a universally adaptable, precise, and efficient TLA workflow for patients diagnosed with malignant liver tumours.

## Supplementary Information

Below is the link to the electronic supplementary material.

Supplementary file1 (DOCX 24 KB)
