# Supplementary material for: Prospective Registry Study on Thermal Liver Ablation of Primary and Secondary Liver Tumours Named the A-IMAGIO Study
Source: Cardiovasc Intervent Radiol. 2025 Jun 23;48(8):1193–9. doi: 10.1007/s00270-025-04093-9 (PMC12325461; doi:10.1007/s00270-025-04093-9)
Supplement: Supplementary file 1 — Supplementary file1 (DOCX 24 KB) [file 270_2025_4093_MOESM1_ESM.docx]

**Supplementary 1**

Table 1. Detailed overview of prospectively collected parameters.

| **Patient characteristics** | - Demographics - Underlying liver disease - Oncological history - Prior (hepatic) tumour treatment - Type of liver tumour (i.e. primary or secondary) |
| --- | --- |
| **Diagnostics** | - Laboratory tests - Diagnostic imaging |
| **Tumour characteristics** | - New/residual - Dimensions - Anatomical location - Tumour stage - Number of hepatic tumours |
| **Procedure related information** | - Procedure technique - Needle guidance - Supporting software - Type of anaesthesia - Technical success - Per-procedural complications |
| **Follow-up** | - Time of follow-up - Imaging modality - Laboratory tests - Local recurrence/ new lesions - (extrahepatic) progression of disease - Complications - Supplementary oncological treatment - 6-months, 1-year, 2-year and 5-year overall survival |
